# IR-780 Dye as a Sonosensitizer for Sonodynamic Therapy of Breast Tumor

**DOI:** 10.1038/srep25968

**Published:** 2016-05-13

**Authors:** Yekuo Li, Qunfang Zhou, Zhiting Deng, Min Pan, Xin Liu, Junru Wu, Fei Yan, Hairong Zheng

**Affiliations:** 1 Department of Ultrasound, Guangzhou General Hospital of Guangzhou Military Command, Guangzhou, China; 2 Paul C. Lauterbur Research Center for Biomedical Imaging, Institute of biomedical and Health Engineering, Shenzhen Institutes of Advanced Technology, Chinese Academy of Sciences, Shenzhen, China; 3 Department of Physics, University of Vermont, Burlington, VT 5405-0160, USA.; 4 Shenzhen Key Laboratory of Nanobiomechanics, Shenzhen Institutes of Advanced Technology, Chinese Academy of Sciences, Shenzhen, China

## Abstract

Sonodynamic therapy (SDT) has become a new modality for cancer therapy through activating certain chemical sensitizers by ultrasound (US). Discovery and development of novel sonosensitizers are attracting extensive attentions. Here, we introduce IR-780 iodide, a lipophilic heptamethine dye with a peak optical absorption of 780 nm wavelength, which can function as SDT agents for breast cancer treatment. The *in vitro* cellular uptake, cell viability, and the generation levels of reactive oxygen species (ROS) were examined by using 4T1 breast cancer cells incubated with various concentrations of IR-780 followed by US irradiation. Our results showed a dose- and time-dependent cellular uptake of IR-780 iodide in 4T1 cancer cells. Significant lower viabilities and more necrotic/apoptotic cells were found when these cancer cells were treated with IR-780 iodide with US irradiation. Further analyzing the generation of ROS demonstrated significant increase of ^1^O_2_ level and H_2_O_2_, but not ⋅OH in the SDT-treated cells. The *in vivo* anti-tumor efficacy of SDT with IR-780 revealed significant tumor growth inhibition of xenografts of 4T1 cancer cells; it was further confirmed by histological analysis and TUNEL staining. Our results strongly suggest that SDT combined with IR-780 may provide a promising strategy for tumor treatment with minimal side effects.

Sonodynamic therapy (SDT) first discovered by Yumita *et al*.^1^, which utilizes low intensity ultrasound (US) to activate a group of photosensitive materials called sonosensitizer and to enhance their cytotoxic effects to targeted cells, is emerging as a promising cancer treatment therapy^2^. Different from photodynamic therapy (PDT), SDT uses non-thermal effects of ultrasound combined with a sonosensitizer. SDT has at least two decided advantages over PDT: (i) US as a mechanical wave can penetrate into deeply seated human soft tissue; (ii) US can be precisely focused on a specific tumor site and effectively activate the sonosensitizer. It has also been demonstrated that SDT can accurately kill the cancer cells with minimal damages to their neighboring normal cells^3^,^4^,^5^.

The exact mechanisms involved in activating sonosensitizers by US irradiation are not fully understood. It has been widely suggested that several possible mechanisms may be involved: (i) Inertial cavitation generated by collapsing microbubbles under excitation of high amplitude US and its concurrently happening sonochemical reactions^6^,^7^; (ii) generation of free radicals either by direct thermolysis or reactions with H^+^/OH^−^ from the thermolysis of water during SDT. The resulting free radicals subsequently produce peroxyl and alkoxyl radicals through reaction with oxygen, resulting in the chain peroxidation of membrane lipids and tumor cell damage^8^; (iii) the sonosensitizers can absorb US energy and be activated when they were exposed to US. The received energy by sonosensitizers would be released when they return to the ground state from the excited state. Oxygen molecules, when they acquire the released energy, will change into excited-singlet-state oxygens (a highly reactive agent) and initiate a series of oxidization reactions which eventually lead to the irreparable cellular damage^9^; (iv) ultrasound-induced apoptosis, i.e., the programmed death pathway will be also activated when enough reactive oxygen species (ROS) are generated in tumor cells by US irradiation^10^,^11^,^12^,^13^. It has been demonstrated that the nano-sized TiO_2_ powder is an effective sonosensitizer; various kinds of ROS can be generated when it was irradiated by US^14^. To date, more and more sonosensitizers are found and applied to the cancer treatment^15^. Some photosensitizers including porphyrin derivatives and phthalocyanins attract the most extensive attentions as sonosensitizers^16^,^17^.

IR-780 iodide, a lipophilic cation heptamethine dye (the molecular structure is shown in Fig. 1) with the peak optical absorption at 780 nm wavelength, has emerged as a potential fluorescent probe for *in vivo* tumor imaging due to its high fluorescence intensity, excellent stability and preferential accumulation in the tumor^18^. In addition, IR-780 can be used for photothermal therapy (PTT) because of its strong absorption at near-infrared wavelengths^19^. Recently, the PDT application of IR780 has also been reported^20^. In this study, we investigated the feasibility of IR-780 as a sonosentizer in the SDT. To our best knowledge, it is the first report about the application of SDT using IR-780 as a sonosensitizer for treatment of breast cancer.

## Materials and Methods

### Materials

The heptamethine cyanine dye IR-780 iodide, Hydroxyphenyl fluorescein solution (HPF) and DMSO were purchased from Sigma-Aldrich (St. Louis, MO, USA). Cell Counting Kit-8 (CCK-8) was obtained from Dojindo Laboratories (Tokyo, Japan). Annexin V-FITC Apoptosis Kit was purchased from Biovision (Biovision, USA). MitoSOX^TM^ Red mitochondrial superoxide indicator and 5-(and-6)-chloromethy-2′,7′-dichlorodihydroflurescein diacetate, acetyl ester (CM-H2DCFDA) were purchased from invitrogen (Eugene, Oregon, USA). The *in situ* Cell Death Detection Kit was obtained from Roche (Mannheim, Germany). Murine breast cancer 4T1 cell line was purchased from American Type Culture Collection. Female BALB/c mice (6 to 10 week old) were purchased from Guangdong Medical Experimental Animal Center (Guangzhou, China). All other reagents were of analytical grade.

### *In vitro* cellular uptake of IR-780 iodide

IR-780 iodide was dissolved in DMSO and diluted with PBS in final concentration of 100 mM for *vitro* experiment. For cellular uptake study, 4T1 cells (2 × 10^6^ cells/dish) were seeded in 35 mm diameter dishes and cultivated for 24 h for full adhesion. The media were replaced with the fresh media containing 4 μM, 10 μM or 16 μM of IR-780 iodide. The medium containing the same volume PBS were used as the control. After 1, 2 or 3 h, the cells were washed with PBS for 3 times and harvested with trypsin digestion. After that, the cells were detected by flow cytometry (BD FACSCanto II, USA).

To further determine the cellular distribution and retention of IR-780 in 4T1 cells, a laser confocal microscopy (Leica TCS SP5, Wetzlar, Germany) was used. Briefly, 4T1 cells were seeded onto 8-well chambered cover-glass at a density of 2 × 10^4^/well (0.4 mL). 24 h later, the medium was changed with fresh medium containing 4 μM, 10 μM or 16 μM of IR-780 iodide. After 1 h incubation, the cells were washed and fixed with 4% paraformaldehyde solution for 20 min, then stained with Hoechst 33258 for 5 min. The cells were examined under a confocal laser scanning microscope.

### *In vitro* cytotoxicity of IR-780 iodide with US irradiation

The cell cultivation and IR-780 treatment were the same as stated above. After washing cells for 3 times, 2 mL of fresh complete medium were added into each dish. All of groups were exposed to US irradiation using a sonicator device purchased from Tianjin Tianshi Technology Company (Tianjin, China). The *in vitro* US experiment instrument was showed in Supplementary Fig. 1. Briefly, the transducer was immerged in a sink filled with degassed water. There was a dish holder on the water surface to fix the cell dish which was not fully immersed in the tank where the transducer is located. The distance between the transducer and the dishes was kept at 3 cm. US irradiation was performed at the spatially and temporally averaged intensity of 1.5 W/cm^2^ (frequency: 1 MHz; duty cycle: 50%, pulse repetition frequency: 1 Hz) for 20 s or 40 s, respectively. After US treatment, the cell viability was assessed by CCK-8 detection kits. The absorbance at 450 nm was determined using a multimode plate reader (Synergy™4, BioTek, VT, USA). For further assess of apoptosis induced by US and IR-780, the 20-sencond-US-treated cells were stained with FITC-conjugated Annexin V and propidium iodide for 15 min and analyzed by flow cytometry (BD Accuri C6, USA).

### Detection of ROS levels

The intracellular ROS were detected after the cells were treated with US and IR-780. As for ^1^O_2_ detection, the probe reagents were dissolved with DMSO at the concentration of 5 mM, and then diluted with PBS at the final concentration of 5 μM for experiment use. 4T1 breast cancer cells (1 × 10^6^/dish) were seeded in 35 × 35 mm dishes and cultivated for 24 h for full adhesion. The medium was replaced by the fresh medium containing 10 μM of IR-780 iodide. As a control, no IR-780 was used. After being incubated for 3 h, the cells were washed and added with the prepared probe solution. Then cells were treated with US irradiation at 1.5 W/cm^2^ for 20 s. After treatment, the cells were harvested, and the fluorescence intensity was measured using a multimode plate reader at the excitation wavelength of 510 nm and emission wavelength of 580 nm. Relative fluorescence units (RFU) of samples were calculated and normalized to the untreated cells. As for the detections of H_2_O_2_ and ⋅OH, CM-H2DCFDA (final concentration of 7.9 μM) and HPF (final concentration of 10 μM) were used, respectively. Similar procedures were performed as stated above. The fluorescence intensity of CM-H2DCFDA was determined at the excitation wavelength of 490 nm and emission wavelength of 525 nm. The fluorescence intensity of HPF was measured at the excitation wavelength of 490 nm and emission wavelength of 515 nm.

### Effect analysis of active oxygen scavengers

The effects of active oxygen scavengers were detected according to the previous report^21^. Briefly, the cell cultivation were the same as stated above. 10 μM of IR-780 iodide was added and incubated for 3 h. After washing out the free IR-780 dye, 2 mL of fresh complete medium were added into each dish. As the control, no IR-780 was used for these cells. Before ultrasound irradiation, histidine (10 mM), mannitol (100 mM) or SOD (100 ug/mL) were added to the cells and US irradiation was performed for 20 s. After that, cell damage induced by insonation in the presence and absence of IR-780 with active oxygen scavengers was detected by staining of the cells with Trypan Blue dye as described previously^1^.

### *In vivo* imaging analysis

All animal studies were approved by the Institute’s Animal Care and Use Committee of Shenzhen Institutes of Advanced Technology, Chinese Academy of Sciences. The methods were carried out in accordance with the approved guidelines. 6- to 8-week-old female BALB/c mice (five per cage) were housed on a 12:12 light: dark cycle with free access to food and water. 4T1 breast carcinoma cells (1 × 10^6^/each) were injected into the right flank of mice. When mice had developed palpable tumors 6 days after tumor cell injection (about 5 mm in diameter), 0.1 mL 800 μg/mL of IR-780 was injected into the tumor. Images and fluorescence semi-quantitative analysis of IR-780 were taken at 0.1 h, 0.5 h, 3 h, 6 h after injection using the *ex*/*in vivo* imaging system (CRi maestro, USA) with a 704 nm excitation wavelength and a 740 nm filter to collect the fluorescence signals of IR-780. 48 h later, the mice were sacrificed and tissue organs were collected for imaging and biodistribution analysis.

### Anti-tumor efficacy of SDT with IR-780

In the *in vivo* experiments, we set up the experiment instrument as shown in Supplementary Fig. 2. Briefly, the transducer was immersed in a degassed water-filled sink. On the surface of water, there is a mouse supporting plate with a hole (15 mm diameter) at the center of the plate. The mouse was placed on the plate and the tumor was positioned into the hole. Tumor-bearing mice were randomly divided into 4 groups (5 animals each group). Mice were intratumorally injected with 100 μL PBS, 100 μL PBS + US, 80 μg of IR-780 (in 100 μL solution), or 80 μg of IR-780 + US. US irradiation was performed at 2 W/cm^2^ for 4 min. The day when mice were received with US treatment was designated as day 0. The tumor volumes and body weights of the mice were recorded. The tumor volumes were calculated by the following formula: Tumor volume (mm^3^) = axial length (mm) × (lateral axial length)^2^ (mm^2^)/2.

### Histological analysis

12 tumor bearing mice (3 mice each group) were sacrificed by standard decapitation 48 h later after US treatments. The tumors were harvested, fixed with formalin and embedded in paraffin. The tumor sections (8 μm) were cut with a paraffin slicing machine (Leica, RM223, Germany) and stained with hematoxylin and eosin (H&E). For assessment of apoptosis, terminal deoxynucleotidyl transferase-mediated dUTP nick end labeling (TUNEL) was carried out with an *in situ* Cell Death Detection Kit according to the manufacturer’s protocol. Cell apoptosis index was determined by calculating the percentage of positively stained cells for all cells from six randomly chosen fields/sections at × 400 magnification.

### Statistical analysis

The results were expressed as the mean ± standard deviation (S.D). Comparisons between cell fluorescence intensities in the uptake experiments were compared using two-way repeated ANOVA test. Data analyses including cell viability, *in vitro* apoptosis/necrosis ratios, levels of ROS, *in vivo* fluorescence intensities and apoptotic index in tumor sections were performed with one-way ANOVA test. Tumor volumes and mouse body weights were compared using a Kruskal-Wallis test followed by the Manne-Whitney test. The differences were considered to be significant for ^*^P < 0.05, and to be very significant for **P < 0.01.

## Results

### Cellular uptake of IR-780 iodide

**Figure 2:**
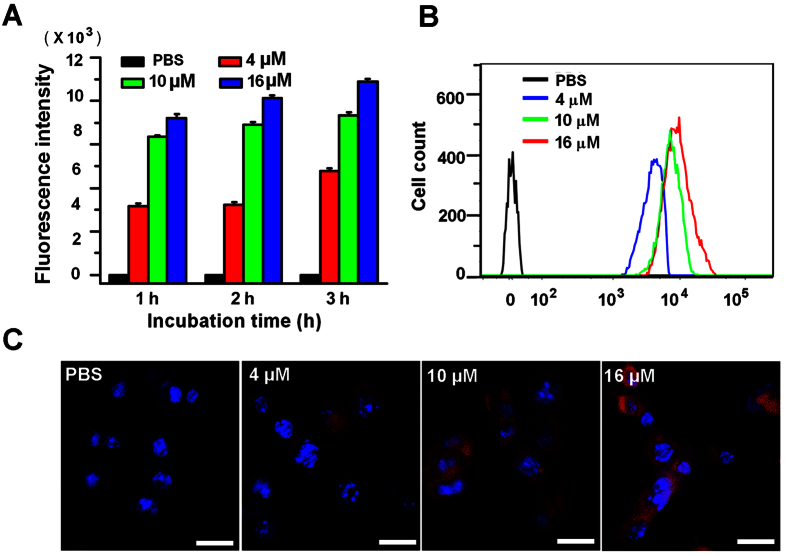
The cellular uptake of IR-780 iodide into 4T1 breast cancer cells. (**A**) The mean fluorescence intensities of 4T1 breast cancer cells incubated with PBS, 4 μM, 10 μM or 16 μM of IR-780 for 1, 2, or 3 h were detected by flow cytometry. (**B**) Representative fluorescence intensity curves for IR-780-incubated cells (for 1 h) by flow cytometry revealed a right shift when compared with the control cells. (**C**) Representative fluorescence micrograph of 4T1 breast cancer cells after incubated with PBS, 4 μM, 10 μM or 16 μM of IR-780 for 1 h. Red stands for IR-780 and blue stands for Hoechst-stained nuclei. (Scale bar = 20 μm).

The uptake of 4T1 cancer cells were investigated with flow cytometry and confocal laser scanning microscope. Results were showed in Fig. 2. Figure 2A demonstrated there were obvious dose- and time-dependence for cellular uptake of IR-780 iodide. At all three examined time points, the fluorescence intensities of the cells incubated with IR-780 iodide increased with an increase of IR-780 concentrations. After 1 h, the fluorescence intensity of the cells incubated with 10 μM or 16 μM of IR-780 iodide were 2.01-fold and 2.27-fold higher than that of the cells with 4 μM of IR-780 iodide, respectively. Similar trends can be found in these cells incubated for 2 or 3 h. Also, stronger fluorescence intensities might be found in these cells with longer incubation time at the same dye dose, indicating the time-dependence of cellular uptake. Figure 2B showed the significant right shift of the fluorescence curves with the increasing dye concentrations after 1 h cell incubation. The results were further confirmed by a laser confocal microscopy, indicating the gradual increasing cellular uptake with the increase of IR-780 doses (Fig. 2C).

### *In vitro* sonotoxic effect with IR-780 iodide on 4T1 cells

**Figure 3:**
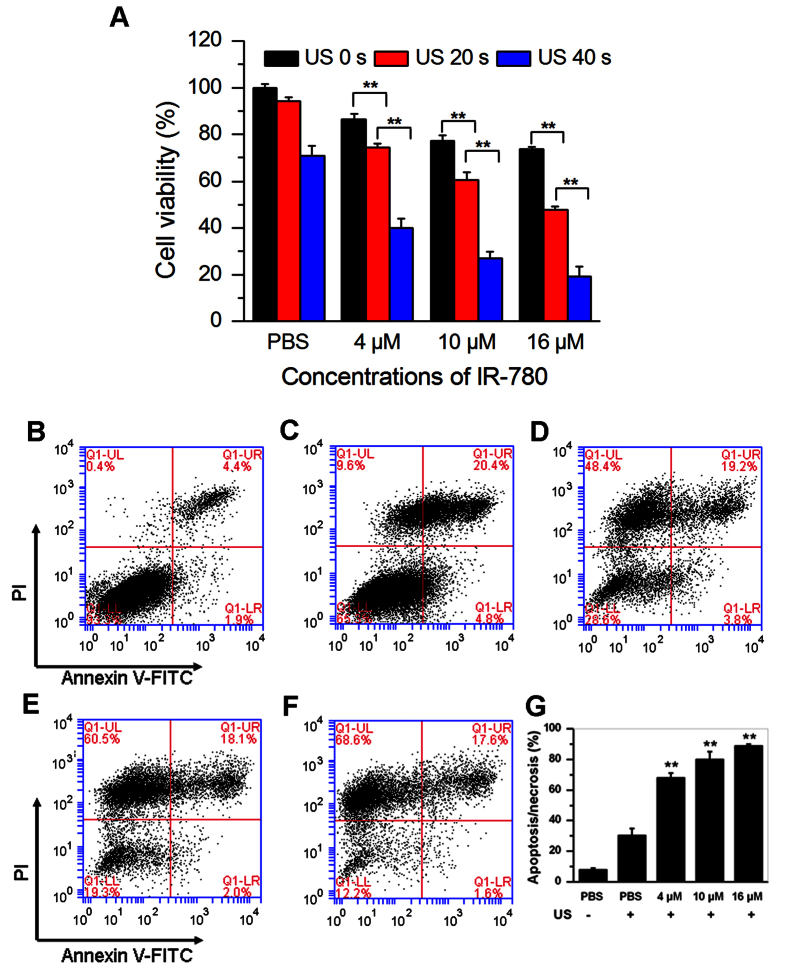
*In vitro* sonotoxic effect with IR-780 iodide on 4T1 cells. (**A**) Cell viability analysis of 4T1 breast cancer cells incubated with PBS, 4 μM, 10 μM or 16 μM of IR-780 followed by US for 0, 20, or 40 s after 24 h later, the cell viability of 4T1 breast cancer cells were detected by CCK-8 kit. (**B–F**) Flow cytometry analysis of tumor cell apoptosis and necrosis after 4T1 breast cancer cells were incubated with PBS (**C**), 4 μM (**D**), 10 μM (**E**) or 16 μM (**F**) of IR-780 followed by US for 20 s 24 h later, the cell viability of 4T1 breast cancer cells were stained with Annexin V and PI, followed by detecting with flow cytometry. The untreated cells were used for a control (**B**). (**G**) The dead cells including apoptotic and necrotic cells were quantified.

Figure 3A showed the viability of the tumor cells received with US irradiation at different time for three different IR-780 iodide doses. Under the condition without US, treatment of tumor cells had minor inhibition effect on cell viability with 86.45 ± 1.12%, 77.19 ± 0.56%, or 73.35 ± 1.26% at 4, 10 or 16 μM dye concentrations, respectively. Only US irradiation for 20 s had not obvious effect on cell viability, but longer US irradiation for 40 s could observe some inhibitive effect on tumor cell viability. It was notable that significant growth inhibition effects on cell viabilities could be seen when IR-780 iodide plus US was used. The tumor cells treated with 4 μM of IR-780 with US showed significantly lower cell viability (74.21 ± 1.96% and 39.99 ± 0.81% of cell viability for 20 s and 40 s US irradiation, respectively) than that treated with 4 μM of IR-780 only (86.45 ± 1.12%) (P < 0.01). The higher concentrations of the IR-780 iodide or longer US duration were used, the better inhibitory effect on tumor cell growth would be. The survival cells decreased to 47.75 ± 0.42% and 19.25 ± 1.03% for 20 s and 40 s US duration when the cells treated with 16 μM of IR-780.

In order to detect whether IR-780 can induce apoptosis of tumor cells under US exposure, 4T1 breast tumor cells were treated with various concentrations of IR-780 and received US irradiation for 40 s. Apoptosis was determined by flow cytometry of the staining cells with Annexin V-FITC/PI. The results demonstrated that IR-780 plus US induced significant tumor cell apoptosis/necrosis compared with the control groups (Fig. 3B–F). There were 67.83 ± 3.16%, 80 ± 4.97% or 88.87 ± 0.97% apoptosis/necrosis tumor cells when the concentrations of IR-780 were 4 μM, 10 μM or 16 μM, respectively. Only 7.93 ± 1.08% and 30.5 ± 4.19% apoptosis/necrosis tumor cells were found in the untreated cells and the only US treated cells (Fig. 3G). These results indicated that IR-780 could act as a potential sonosensitizer for SDT.

### Levels of ROS generation

**Figure 4:**
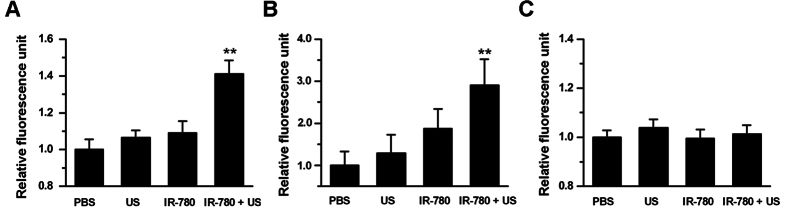
The ROS analysis of 4T1 breast cancer cells received SDT with IR-780. 4T1 breast cancer cells were incubated with PBS or 16 μM of IR-780, and then these cells received with US for 40 s or without US irradiation. 24 h later, the levels of ^1^O_2_ (**A**), H_2_O_2_ (**B**) and ⋅OH (**C**) in these cells were detected.

We further investigated whether IR-780 could induce the generation of ROS in the tumor cells after US irradiation. Figure 4A demonstrated that the tumor cells treated with IR-780 plus US caused significant increased level of ^1^O_2_, with 1.55-fold, 2.29-fold and 2.91-fold higher fluorescence intensities than that of the tumor cells treated with IR-780, only US or PBS control, respectively (P < 0.01). Figure 4B demonstrated there had a significant increase of H_2_O_2_ in the tumor cells treated with IR-780 plus US. There were 1.29-fold, 1.33-fold and 1.41-fold higher fluorescence intensities compared with the tumor cells treated with IR-780, only US or PBS control, respectively (P < 0.01). No significant increase of ⋅OH levels was found in the tumor cells treated with IR-780 plus US (P > 0.05)(Fig. 4C).

### Effect of active oxygen scavengers

**Figure 5:**
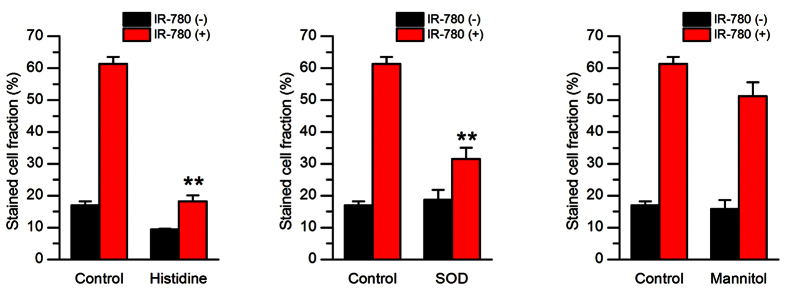
Effect of active oxygen scavengers on ultrasound cell damage by SDT with IR-780. 4T1 breast cancer cells were incubated with PBS or 10 μM of IR-780. Histidine (10 mM), SOD (100 μg/mL) or mannitol (100 mM) was added to the cells before US exposure. Cell damage was detected by Trypan Blue staining. The stained cell fraction was expressed the percent of blue stained cells in all of the examined cells.

Since IR-780 could induce the generation of ROS in the tumor cells after US irradiation, we further used active oxygen scavengers to confirm the effects. The results were shown in Fig. 5. Obviously, cell damage by US alone was partly inhibited by histidine, but its enhancement by IR-780 was greatly inhibited (P < 0.01). SOD had no effect to inhibit the cell damage by US alone, while it could also decreased the cell damage by US + IR-780 (P < 0.01), which was similar with the effect of histidine. Mannitol had no effect in decreasing the cell damage of US with or without IR-780 (P > 0.05).

### *In vivo* imaging of IR-780 in tumor-bearing mice

**Figure 6:**
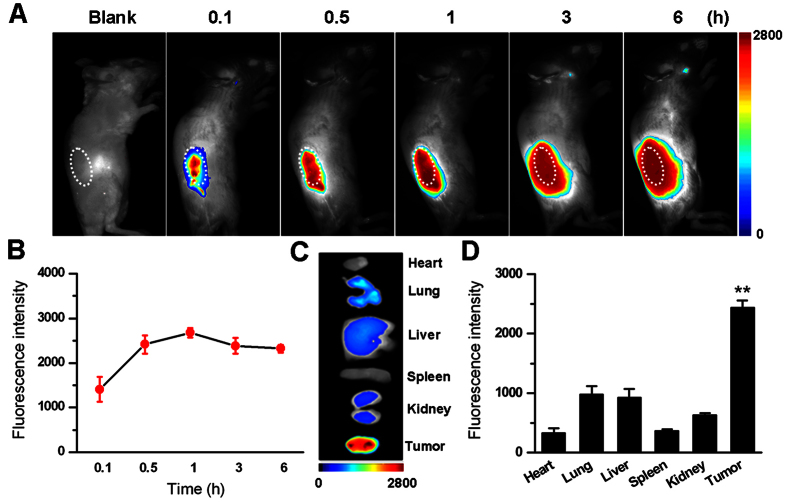
The *in vivo* fluorescence imaging of tumor-bearing mice before and after injected with IR-780. (**A**) The representative fluorescence micrograph of tumor-bearing mice before and 0.1, 0.5, 1, 3, or 6 h after injected with IR-780. White cycles for tumor sites. (**B**) The mean fluorescence intensities of tumors were quantified, showing a peak value after 1 h injection of IR-780. (**C**) The representative fluorescence micrograph of heart, lung, liver, spleen, kidney and tumor from tumor-bearing mice injected with IR-780. (**D**) The mean fluorescence intensities of these tissues were quantified, showing the highest fluorescence intensity in tumor.

In order to determine the optimal time for *in vivo* SDT using IR-780, we examined the tissue permeability and distribution of IR-780 in the tumor-bearing mice by a whole animal NIR imaging system. Figure 6A indicated the fluorescence signals increased gradually as IR-780 diffusing in the tumor tissue, reaching a maximum tumor coverage area after 1 h. A longer time (>6 h) would result in the permeability of IR-780 into the peripheral normal tissues. The quantitative values of fluorescence intensities at different time were presented in Fig. 6B, revealing that the fluorescence intensity in the tumors could almost achieve the maximum level after 1 h. The tumor localization of IR-780 was still dominant even if 48 h later (Fig. 6C,D).

### *In vivo* therapeutic efficacy of IR-780 plus US

**Figure 7:**
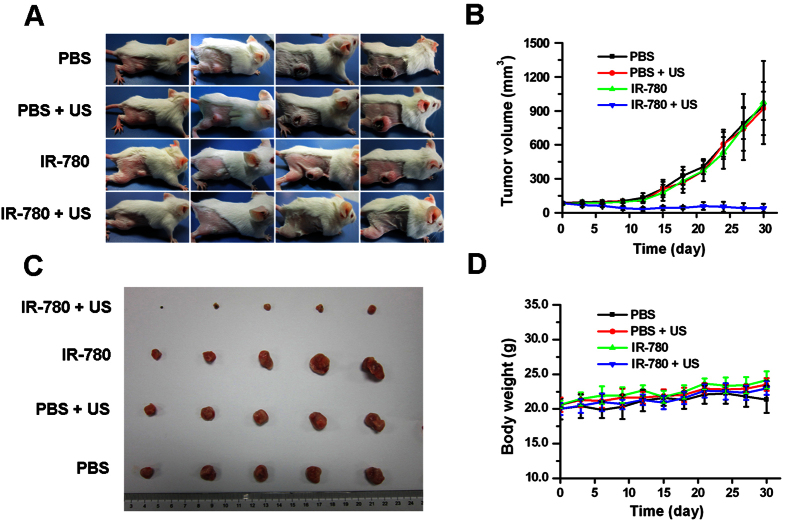
The tumor growth can be inhibited by SDT with IR-780. (**A**) The representative photographs of tumor-bearing mice before and after treated for 11, 23 or 30 days. (**B**) The tumor growth curves of tumor-bearing mice treated by SDT with IR-780. (**C**) The photograph of tumors removed from mice after treatment (30 days). (**D**) The body weight changes of tumor-bearing mice before and after treatment.

Next, we tested the *in vivo* efficacy of IR-780 as a sonosensitizer. Figure 7A showed the representative tumor-bearing mice at day 0, day 11, day 23 and day 30. The tumor growth curves were presented in Fig. 7B. Obviously, mice received with IR-780 plus US showed a significant tumor growth inhibition (P < 0.01), with the mean tumor size of 40.7 ± 36.93 mm^3^ at day 30. By contrast, there were not significant inhibition effects on tumor growth for IR-780 group or only US group (P > 0.05), with the mean tumor size of 973.06 ± 367.98 mm^3^ or 921.62 ± 234.39 mm^3^ at day 30, respectively. Figure 7C showed all of the tumors removed from four groups of tumor-bearing mice at day 30, demonstrating a dramatic decrease of tumor sizes when they received SDT with IR-780. No significant changes of body weight of mice were observed over the course of the mouse experiment (Fig. 7D). Additionally, we measured the surface temperature of the tumors through an electronic thermometer while the tumors were irradiated by US. It was demonstrated that the temperature of the tumors of both groups of animals receiving US did not have obvious increase, excluding the thermal effects by US irradiation in our experiments (data not shown).

### Histological analyses

**Figure 8:**
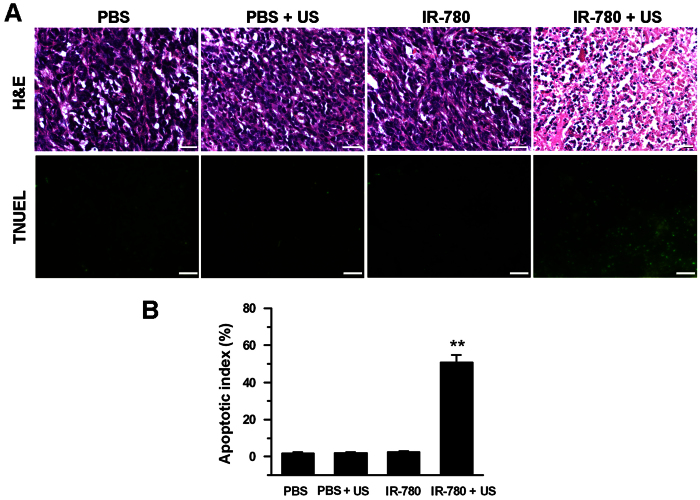
Histological analysis of tumors after treated SDT with IR-780. (**A**) H&E and TUNEL staining analysis of tumors from mice received with PBS, PBS + US, IR-780 and IR-780 + US. A significant tumor necrosis (upper row) and more apoptotic cells (bottom row, green) can be found in the tumor treated by IR-780 + US. (**B**) The apoptotic index was determined by calculating the percent of apoptotic cells versus the total cells in the same field. (Scale bar = 40 μm).

The tumor sections from mice after 48 h treatment were stained with H&E and examined under light microscope. A significantly increased degree of cell necrosis could be observed in the tumors receiving IR-780 and US (Fig. 8A, upper row). To elucidate whether SDT with IR-780 induce tumor cell apoptosis, TUNEL staining were performed. Results demonstrated that TUNEL-positive cells (green) were significantly more in the tumors treated using SDT with IR-780, compared with other groups (Fig. 8A, bottom row). Quantitatively, there were 50.9 ± 3.89% apoptotic tumor cells found in the tumors received IR-780 plus US. By contrast, there were 1.8 ± 0.77%, 1.85 ± 0.67% and 2.35 ± 0.57% apoptotic cells found in the tumors received PBS, only US or only IR-780, respectively (P < 0.01) (Fig. 8B).

## Discussion

Currently, it is widely accepted that presence of potential sonosensitizers is one of the most essential factors in SDT. Identification and development of novel sonosensitizers are attracting more and more researchers’ attention. In this study, we found the remarkable toxicity of SDT using IR-780 as a sonosensitizer against 4T1 breast cancer *in vitro* and *in vivo*. To our best knowledge, there is no literature reporting IR-780 agent as a sonosentizer in SDT. Our study provided evidences that IR-780 can function as a sonosentizer in SDT of breast tumor. In fact, some photosensitizers including porphyrin derivatives and phthalocyanins are also found to have great potentials as sonosensitizers and attract extensive attentions^22^. Given the unique advantages of SDT over PDT, there are enough reasons for us to believe that IR-780 can have a promising application in tumor treatment.

In our study, 4T1 breast cancer cells demonstrated excellent uptake efficiency for IR-780 iodide (Fig. 2). It is easy to understand when considering the fact that the lipophilic property of this dye may favor it to effectively cross the membrane into the tumor cells. Also, we found that the free IR-780 iodide and only US had some anti-proliferative activity on 4T1 breast cancer cells (Fig. 3A), demonstrating a proper dye dose and US energy should be optimized for future clinical utilities. Also, several approaches, such as encapsulation of this dye with biocompatible nanomaterials or utilization of local intratumoral administration, may decrease the potential cytotoxicity of IR-780 dye to health cells. It was demonstrated the cytotoxicity from the non-thermal US mainly result from the mechanical stress or sonochemical effects of acoustic cavitation^23^. In our study, SDT with IR-780 mainly led to cell necrosis and late apoptosis (Fig. 3B–G). It is known that PDT can induce apoptosis and necrosis, and necrotic response may be the major cell death pathway in high-dose photosensitizer with PDT^24^. This also can be expected for SDT with IR-780. In the Tsuru’s report, it was proved that SDT with DEG mainly led to necrosis instead of apoptosis^25^. Indeed, various kinds of factors, such as sonosensitizers, tumor cell lines, and US parameters, may affect the ratios of apoptosis or necrosis of tumor cells in SDT^26^.

**Figure 9:**
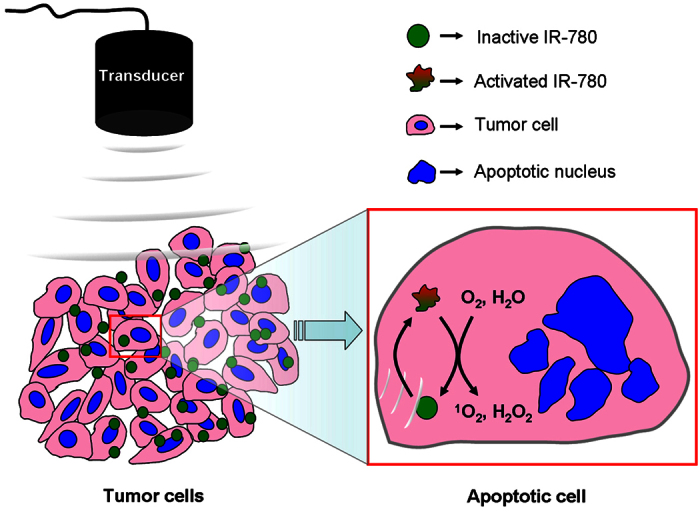
The possible mechanisms of tumor treatment of SDT with IR-780. IR-780 can be uptaken by the tumor cells. Upon receiving US, IR-780 in the tumor cells would receive the US energy and be activated from a ground-state into an excited state. When the excited-stated IR-780 returns to the ground-stated one, the energy could be released; ^1^O_2_ and H_2_O can absorb the released energy and change into ^1^O_2_ and H_2_O_2_. The superfluous ^1^O_2_ and H_2_O_2_ would cause the apoptosis and necrosis of tumor cells.

The generation of ROS plays an important role in SDT of tumor treatment. Our data showed that ^1^O_2_ and H_2_O_2_, but not ⋅OH were found to be an increased generation after 4T1 cancer cells treated with IR-780 and US (Fig. 4A,B). This was confirmed by the sonodynamic cell damage experiments, in which the sonodynamic cell damage can be inhibited by histidine and SOD, but not by mannitol (Fig. 5). The species of ROS induced by SDT were involved in the kinds of sonosensitizers^27^. Previous studies revealed that singlet oxygen was a major source of ROS from porphyrin-derived sensitizers in both PDT and SDT^28^,^29^. Our result was consistent with the SDT with porphyrins^30^,^31^. Thus, a possible mechanism of IR-780 for SDT may be speculated as follows: IR-780 can be uptaken by the tumor cells. Upon receiving US irradiation, IR-780 in the tumor cells would be activated and changed into the excited state from the ground state. Due to the instability of the excited-state IR-780, they would trend to return to the ground state and to release the US energy. Oxygen molecules and H_2_O can acquire the released energy and turn into ^1^O_2_ and H_2_O_2_. With the increase of the concentrations of ^1^O_2_ and H_2_O_2_, the tumor cells would be progressively damaged through oxidative stress-induced cell death pathways (Fig. 9). In fact, the specific mechanisms of SDT are dependent on various factors, including tumor cell models, experimental systems utilized, the type of sonosensitizers and the US exposure parameters (such as frequency and intensity)^32^. It is also difficult to give a universal mechanism of SDT. Our study, at least partly, demonstrated that SDT with IR-780 can kill 4T1 tumor cells through increasing the level of ^1^O_2_ and H_2_O_2_ in these tumor cells.

In this study, we further investigated the efficacy of SDT with IR-780 and demonstrated the great tumor growth inhibition *in vivo* xenografts of 4T1 cancer cells (Fig. 7). An obvious tumor necrosis and more apoptotic tumor cells could be observed in the H&E- or TUNEL-stained microscopic section of tumors received SDT with IR-780 (Fig. 8). The increased apoptosis may contribute to the generation of ROS in the process of IR-780 exposure to US. No obvious adverse effects were observed during our tumor treatments. Our data showed for the first time that SDT with IR-780 significantly inhibited the tumor growth with increased apoptosis and necrosis. Thus provides a promising SDT agent for tumor treatment with minimal side effects.

## Conclusions

Our study demonstrated that IR-780 can act as a sonosensitizer with a great potential for SDT of breast cancer. Since the previous documents have reported its usefulness for PTT and PDT, our data revealed its possible utilization for SDT and provided a promising strategy for breast tumor treatment via US.

## Additional Information

**How to cite this article**: Li, Y. *et al*. IR-780 Dye as a Sonosensitizer for Sonodynamic Therapy of Breast Tumor. *Sci. Rep.* **6**, 25968; doi: 10.1038/srep25968 (2016).

## Supplementary Material

Supplementary Information

## Figures and Tables

**Figure 1:**
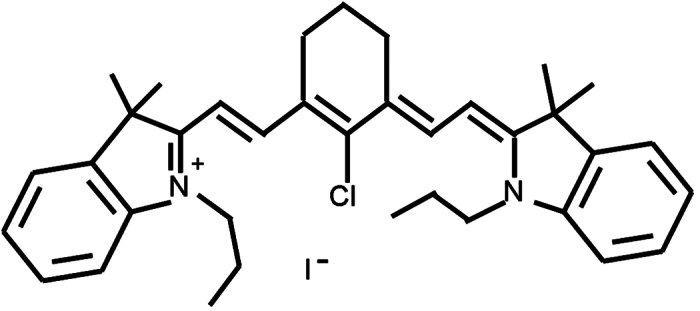
The molecular structure of IR-780 iodide.

## References

[b1] Yumita N., Nishigaki R., Umemura K. & Umemura S. Hematoporphyrin as a sensitizer of cell-damaging effect of ultrasound. Jpn. J. Cancer Res. 80, 219–222 (1989). 2470713 10.1111/j.1349-7006.1989.tb02295.x PMC5917717

[b2] Trendowski M. The promise of sonodynamic therapy. Cancer Metastasis Rev. 33, 143–160 (2014). 24346159 10.1007/s10555-013-9461-5

[b3] Yumita N. . Sonodynamically-induced cell damage with fluorinated anthracycline derivative, FAD104. Cancer Lett. 125, 209–214 (1998). 9566717 10.1016/s0304-3835(97)00515-6

[b4] Harada Y. . Ultrasound activation of TiO_2_ in melanoma tumors. J. Control. Release 149, 190–195 (2011). 20951750 10.1016/j.jconrel.2010.10.012

[b5] Sassaroli E. & Hynynen K. Cavitation threshold of microbubbles in gel tunnels by focused ultrasound. Ultrasound Med. Biol. 33, 1651–1660 (2007). 17590501 10.1016/j.ultrasmedbio.2007.04.018 PMC2078601

[b6] Trendowski M. . The real deal: using cytochalasin B in sonodynamic therapy to preferentially damage leukemia cells. Anticancer Res. 34, 2195–2202 (2014). 24778021

[b7] Wang X., Wang Y., Wang P., Cheng X. & Liu Q. Sonodynamically induced anti-tumor effect with protoporphyrin IX on hepatoma-22 solid tumor. Ultrasonics 51, 539–546 (2011). 21329954 10.1016/j.ultras.2010.12.001

[b8] Rosenthal I., Sostaric J. Z. & Riesz P. Sonodynamic therapy-a review of the synergistic effects of drugs and ultrasound. Ultrason. Sonochem. 11, 349–363 (2004). 15302020 10.1016/j.ultsonch.2004.03.004

[b9] Lagneaux L. . Ultrasonic low-energy treatment: a novel approach to induce apoptosis in human leukemic cells. Exp. Hematol. 30, 1293–1301 (2002). 12423682 10.1016/s0301-472x(02)00920-7

[b10] Wang X. . Role of autophagy in sonodynamic therapy-induced cytotoxicity in S180 cells. Ultrasound Med. Biol. 36, 1933–1946 (2010). 20888686 10.1016/j.ultrasmedbio.2010.06.022

[b11] Song D. . Study of the mechanism of sonodynamic therapy in a rat glioma model. Onco. Targets Ther. 7, 1801–1810 (2014). 25336971 10.2147/OTT.S52426 PMC4199795

[b12] Goertz D. E. . Antitumor effects of combining docetaxel (taxotere) with the antivascular action of ultrasound stimulated microbubbles. PLoS One 7, 1–11 (2012). 10.1371/journal.pone.0052307 PMC3527530 23284980

[b13] Yoshida T. . Combination of doxorubicin and low-intensity ultrasound causes a synergistic enhancement in cell killing and an additive enhancement in apoptosis induction in human lymphoma U937 cells. Cancer Chemother. Pharmacol. 61, 559–567 (2008). 17505825 10.1007/s00280-007-0503-y

[b14] Wang J. . Detection and analysis of reactive oxygen species (ROS) generated by nano-sized TiO_2_ powder under ultrasonic irradiation and application in sonocatalytic degradation of organic dyes. Ultrason. Sonochem. 18, 177–183 (2011). 20684888 10.1016/j.ultsonch.2010.05.002

[b15] Chen H. . Recent progress in development of new sonosensitizers for sonodynamic cancer therapy. Drug Discov. Today 19, 502–509 (2014). 24486324 10.1016/j.drudis.2014.01.010

[b16] Sadanala K. C. . Sono-photodynamic combination therapy: a review on sensitizers. Anticancer Res., 34, 4657–4664 (2014). 25202041

[b17] Wang X. . Sonodynamic and photodynamic therapy in advanced breast carcinoma: a report of 3 cases. Integr. Cancer Ther. 8, 283–287 (2009). 19815599 10.1177/1534735409343693

[b18] Zhang C. . Sentinel lymph node mapping by a near-infrared fluorescent heptamethine dye. Biomaterials 31, 1911–1917 (2010). 19963270 10.1016/j.biomaterials.2009.11.061

[b19] Peng C. L. . Multimodal image-guided photothermal therapy mediated by 188Re-labeled micelles containing a cyanine-type photosensitizer. ACS Nano. 5, 5594–5607 (2011). 21671580 10.1021/nn201100m

[b20] Wilk K. A. . Photo-oxidative action in MCF-7 cancer cells induced by hydrophobic cyanines loaded in biodegradable microemulsion-templated nanocapsules. Int. J. Oncol. 41, 105–116 (2012). 22552322 10.3892/ijo.2012.1458

[b21] Umemura S., Yumita N., Nishigaki R. & Umemura K. Mechanism of cell damage by ultrasound in combination with hematoporphyrin. Jpn. J. Cancer Res. 81, 962–6 (1990). 2172198 10.1111/j.1349-7006.1990.tb02674.x PMC5918111

[b22] Xu H. N. . Preparation and sonodynamic activities of water-soluble tetra-α-(3-carboxyphenoxyl) zinc(II) phthalocyanine and its bovine serum albumin conjugate. Ultrason. Sonochem. 22, 125–131 (2015). 24927903 10.1016/j.ultsonch.2014.05.019

[b23] Gao Z. . Sonodynamic therapy inhibits angiogenesis and tumor growth in a xenograft mouse model. Cancer Lett. 335, 93–99 (2013). 23402818 10.1016/j.canlet.2013.02.006

[b24] Wong T. W., Tracy E., Oseroff A. R. & Baumann H. Photodynamic therapy mediates immediate loss of cellular responsiveness to cytokines and growth factors. Cancer Res. 63, 3812–3818 (2003). 12839978

[b25] Tsuru H., Shibaguchi H., Kuroki M., Yamashita Y. & Kuroki M. Tumor growth inhibition by sonodynamic therapy using a novel sonosensitizer. Free Radic. Biol. Med. 53, 464–472 (2012). 22588110 10.1016/j.freeradbiomed.2012.04.025

[b26] Sun X. . Real-time detection of intracellular reactive oxygen species and mitochondrial membrane potential in THP-1 macrophages during ultrasonic irradiation for optimal sonodynamic therapy. Ultrason. Sonochem. 22, 7–14 (2015). 25023826 10.1016/j.ultsonch.2014.06.016

[b27] Miyoshi N., Igarashi T. & Riesz P. Evidence against singlet oxygen formation by sonolysis of aqueous oxygen-saturated solutions of hematoporphyrin and rose bengal. The mechanism of sonodynamic therapy. Ultrason. Sonochem. 7, 121–124 (2000). 10909730 10.1016/s1350-4177(99)00042-5

[b28] Castano A. P., Mroz P. & Hamblin M. R. Photodynamic therapy and anti-tumour immunity. Nat. Rev. Cancer 6, 535–545 (2006). 16794636 10.1038/nrc1894 PMC2933780

[b29] Yumita N., Han Q. S., Kitazumi I. & Umemura S. Sonodynamically-induced apoptosis, necrosis, and active oxygen generation by mono-l-aspartyl chlorin e6. Cancer Sci. 99, 166–172 (2008). 17970784 10.1111/j.1349-7006.2007.00653.x PMC11158503

[b30] Yumita N., Umemura S. & Nishigaki R. Ultrasonically induced cell damage enhanced by photofrin II: mechanism of sonodynamic activation. In Vivo 14, 425–429 (2000). 10904876

[b31] Yumita N. . Involvement of reactive oxygen species in sonodynamically induced apoptosis using a novel porphyrin derivative. Theranostics 2, 880–888 (2012). 23082100 10.7150/thno.3899 PMC3475214

[b32] Tachibana K., Feril L. B. & Ikeda-Dantsuji Y. Sonodynamic therapy. Ultrasonics 48, 253–259 (2008). 18433819 10.1016/j.ultras.2008.02.003

