# Supplementary material for: IR-780 Dye as a Sonosensitizer for Sonodynamic Therapy of Breast Tumor
Source: Sci Rep. 2016 May 13;6:25968. doi: 10.1038/srep25968 (PMC4865802; doi:10.1038/srep25968)
Supplement: Supplementary Information [file srep25968-s1.doc]

**IR-780 Dye as a Sonosensitizer for Sonodynamic Therapy of Breast Tumor**

Yekuo Li ^1, *^, Qunfang Zhou ^1, *^, Zhiting Deng ^2^, Min Pan ^2^, Xin Liu ^2^, Junru Wu ^3^, Fei Yan ^2, #^, and Hairong Zheng ^2, 4, #^

^1^ Department of Ultrasound, Guangzhou General Hospital of Guangzhou Military Command, Guangzhou, China

^2^ Paul C. Lauterbur Research Center for Biomedical Imaging, Institute of biomedical and Health Engineering, Shenzhen Institutes of Advanced Technology, Chinese Academy of Sciences, Shenzhen, China

^3^ Department of Physics, University of Vermont, Burlington, VT 5405-0160

^4^ Shenzhen Key Laboratory of Nanobiomechanics, Shenzhen Institutes of Advanced Technology, Chinese Academy of Sciences, Shenzhen, China.

^*^ These authors contributed equally to this work.

E-mail address: [fei.yan@siat.ac.cn](mailto:fei.yan@siat.ac.cn) (F. Yan), [hr.zheng@siat.ac.cn](mailto:hr.zheng@siat.ac.cn) (H. Zheng).


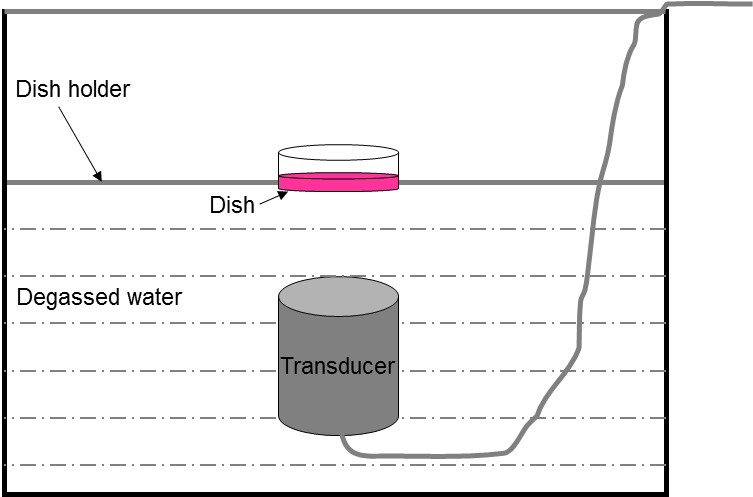


**S-Figure 1** The diagram of the experiment instrumentation used in the in vitro experiments.


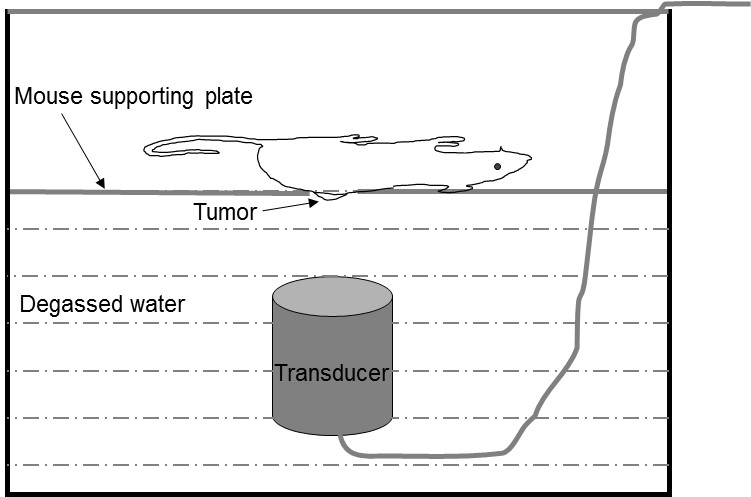


**S-Figure 2** The diagram of the experiment instrumentation used in the in vivo experiments.


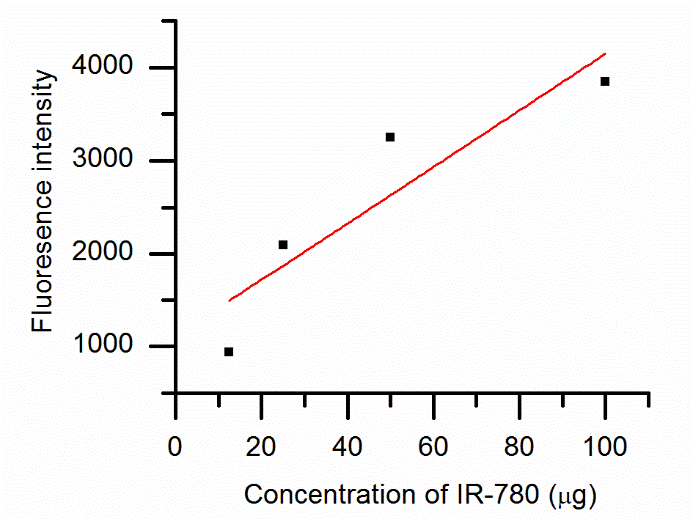


**S-Figure 3** The calibration curve for the IR-780 dye concentrations detected in the NIR imaging system
